# Long term compositional profiling of historical tokaji aszú wines

**DOI:** 10.1038/s41538-025-00468-x

**Published:** 2025-06-14

**Authors:** Ágota Ragyák, Zsófi Sajtos, Edina Baranyai, Elemér László

**Affiliations:** 1 Environmental Analytical Research Group, Department of Inorganic and Analytical Chemistry, Faculty of Science and Technology, University of Debrecen, Egyetem Square 1, H-4032 Debrecen, Hungary; 2 University of Debrecen, Doctoral School of Chemistry, Debrecen, Hungary; 3 Isotope Climatology and Environmental Research Centre (ICER), Institute for Nuclear Research (ATOMKI), Bem tér 18/c, Debrecen, H-4026 Hungary

**Keywords:** Chemistry, Metals

## Abstract

This study examines the elemental composition of unique Tokaji aszú wines produced between 1999 and 2019, representing the longest period for this wine type to date. Twenty-one samples were analyzed using ICP-OES and FTIR techniques. Multivariate statistical analysis revealed significant variations linked to vintage, annual precipitation, sunshine hours, and temperature. Potassium and chromium levels showed strong negative correlations with age, while calcium exhibited a slight increasing trend. FTIR analysis highlighted compositional differences driven by environmental factors, with PCA clustering vintages based on similar weather patterns. These findings emphasize the influence of environmental conditions on the elemental profile of aged aszú wines, offering insights into historical agricultural practices and environmental shifts. The study underscores the value of long-term wine analysis for understanding climate impacts, optimizing future wine production, and preserving the heritage of traditional viticulture. It highlights elemental profiling as a promising tool for historical analysis, environmental assessment, and sustainable viticultural planning.

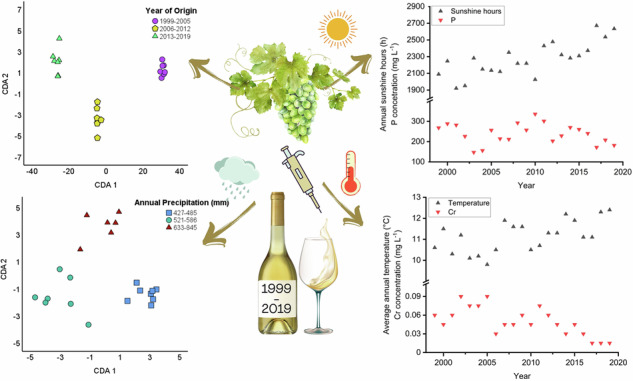

## Introduction

The chemical composition of wine is significantly influenced by various factors, including climate (particularly the meteorological conditions during the growing season), soil properties, geographical features of the vineyard and grape species. Additionally, viticulture management practices and manufacturing processes play a crucial role in determining the qualitative and quantitative parameters of the final product^1–5^. Consequently, each wine can be considered a unique combination of these factors^6^. Among these influences, the geological background is one of the most significant in shaping the chemical profile of the wine, as non-carbon, hydrogen, and oxygen elements present in berries and wine are derived from the soil and rock constituents^7–9^. Soil type is particularly important in many research attempts to find a correlation between the composition and origin of the wine products, even though soil-plant interactions are highly involved. Fingerprinting wine provenance is thus quite challenging owing to the high number of different conditions affecting its composition, still trace elements can be used as authenticity indicators to verify origin^10,11^. Multivariate statistical analysis is used to discriminate wine samples based on their elemental profile in an attempt to link them to their respective origin. The elemental pattern has strong classification potential among samples, particularly when chemometric tools are used to select the most discriminative set of elements^12,13^.

Although the mineral content of wine is relatively low, a substantial body of literature discusses its role in metabolic and biochemical processes^14–18^ as well as the analytical methods for their quantitative determination are quite wide^19–23^.

The Tokaj wine producing region is located in north-eastern Hungary, including around 5.500 hectares of vineyards covering 27 small towns and villages with the commercial center of Tokaj, the town after which the wine region is named. The wine type present in this study is the so-called *aszú*, an intensely sweet and flavored product unique to the Tokaj viticultural district. Aszú belongs to the noble sweet wines, it was described by Louis XIV of France as “Vinum Regum, Rex Vinorum”—the Wine of Kings, King of Wines. It is produced from a variety of white grapes such as hárslevelű, furmint and yellow muscat with the berries left late in the harvest season so they undergo noble rot^24–27^. In this process, *Botrytis cinerea* fungus plays a pivotal role by causing dehydration, thus the concentration of sugars, acids, and flavors within the fruit^28^. According to the traditional process, gathered botrytized berries are crushed and mixed with base wine, then fermented in a slower process due to their higher sugar content. The wine is left to age in tanks or barrels for an extended period up to several years. According to the definition the ‘*3, 4, 5 or 6 puttonyos aszú*’ is a wine with a protected designation of origin (PDO) which is made by pouring new wine, must or new wine in fermentation on the raisined grapes, which is followed by an at least three years long maturation process (two of which take place in barrels). The sugar and sugar-free content is also specified. The denomination *aszú* may only be used with the protected designation of origin “Tokaji”. In viticulture, a “puttony” refers to a traditional unit of measure used historically in Hungary’s Tokaj region and is based on the amount of botrytized grapes added to a barrel of must or wine during the production of *aszú* wines^29–31^. Therefore, the more “puttony” added, the sweeter and more concentrated the resulting wine will be^32^.

The hypothesis of this study is based on the fact that *aszú* wines can be stored for a significantly longer period of time compared to other types due to their unique way of production. The key factors contributing to their longer shelf-life is their high sugar content acting as a natural preservative—inhibiting microbial activity and oxidative processes—as well as the careful fermentation and aging under optimal conditions. Our research group have successfully used the non-perishable honey as archives of information regarding the production processes and the environmental changes due to its ability to retain elemental composition over a longer time period^33,34^. We have analysed honey samples from the last three decades originating from the same nectar-producing area and apicultural specialist. It was found that environmental-related information can be drawn from the change of elemental composition with the age of the measured honeys. *Aszú* wines are also special samples from this context as they are available over a longer time scale; thus, the goal of the present study is to explore their potential for environmental assessment in terms of longevity. The unique feature of this study relies in the type and origin of the samples: wines are provided annually from the same vinery over a span of 20 years for analysis, thus results are not affected by different soil properties, grape cultivation practices or viticultural production methods. If origin verification is a valid approach according to very recent literature data^2,35–38^ then old wines could also be the source of environmental-related information based on their elemental pattern regardless the many circumstances affecting their composition.

## Results

### Elemental composition of wine samples

Since the production of wine is a process based on a number of well-defined steps, the metals present in it can be differentiated according to their natural or anthropogenic origin. The metals’ concentration can significantly affect many parameters, including the flavor, shelf-life and consumability of the wine^11,39^. For instance, the elevated levels of residual Cu(II) have been associated with an accelerated occurrence of oxidative spoilage, culminating in the discoloration of wine, diminishing its freshness and aroma, and the emergence of concentrated sediments and tannins^40^.

However, the inorganic content of wine samples can be affected by the geological and geographical features of the wine region, thus elemental correlation can be used as a characteristic of vintage and origin^41,42^. The average elemental content in the studied wines decreased in the order K > S > P > Mg > Ca > Na > B > Fe > Zn > Al > Mn > Sr > Cu > Ba > Ni > Cr > Pb. A similar tendency was found in Croatian wines by Fiket et al.^5^; however, in Czech wines, the order of the major elements was K > Ca > Mg > P > Na > Fe > Mn^43^. The obtained concentrations for most of the analysed elements are similar to the data reported by Katona et al. for Hungarian aszú wines^44^.

### Comparative analysis of old and recent samples

**Fig. 1:**
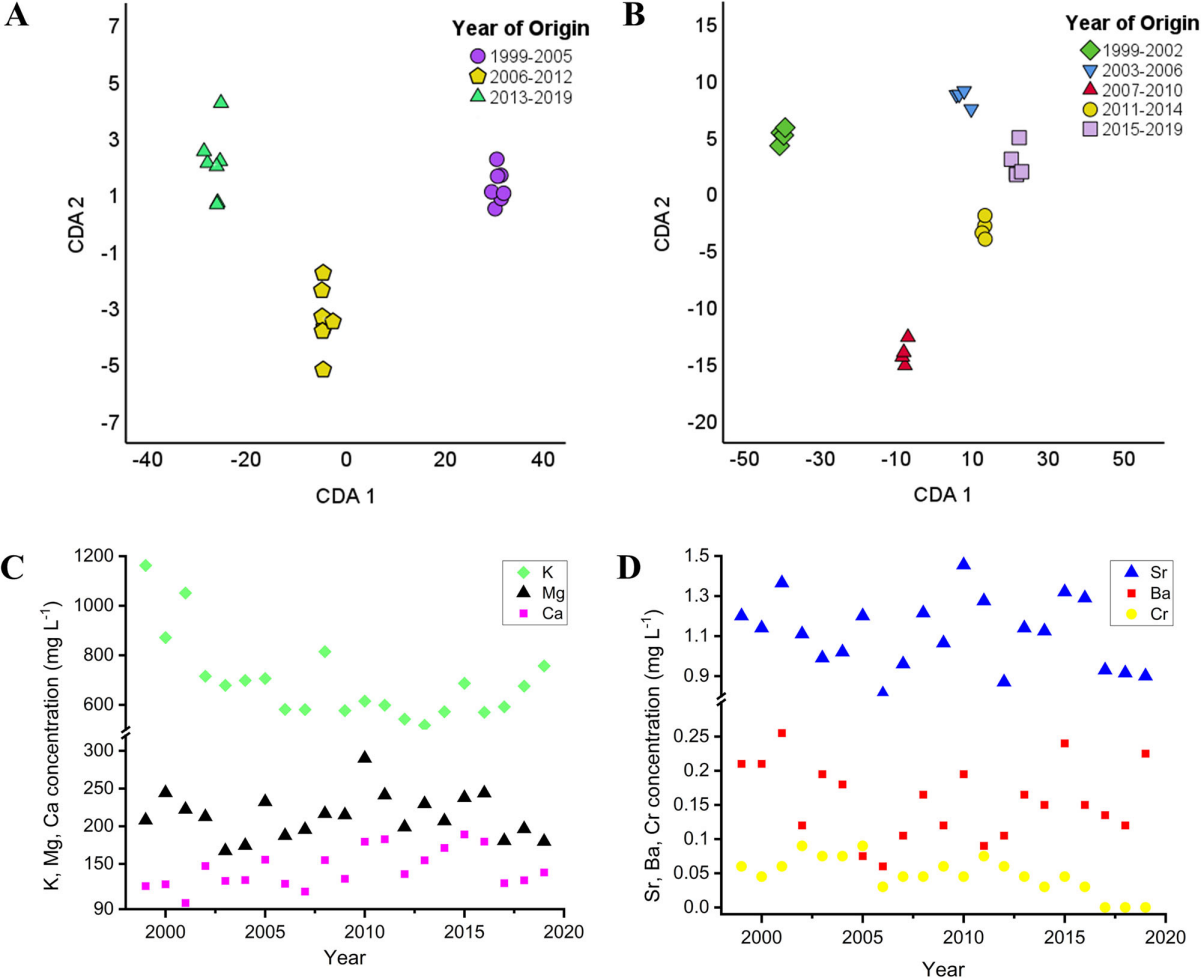
CDA biplot showing group separation of wine samples by vintage (grouped by year). Axes represent canonical functions accounting for variance in elemental composition (Samples are grouped based on years of origin as follows: **A** 1999–2005; 2006–2012; 2013–2019 and **B** 1999–2002; 2003–2006; 2007–2010; 2011–2014; 2015–2019). **C** The concentration of elements in wines plotted against the year of origin through the example of potassium, magnesium, calcium and **D** strontium, barium, chromium. Results are given in mg L^−1^.

Multivariate statistical analysis was carried out on the elemental concentrations of the wines by making groups based on the samples’ year of origin: 1999–2005; 2006–2012; 2013–2019 (Fig. 1A) and 1999–2002; 2003–2006; 2007–2010; 2011–2014; 2015–2019 (Fig. 1B). As indicated in the CDA biplots in Fig. 1A, B, a separation was found considering the elemental data of wines collected in the recent years compared to the older groups. In case of Fig. 1A, the first function accounts for 99.0% of the discriminating ability of the discriminating variables while the second accounts for 1.0%. The canonical correlation values are 0.999 and 0.933, respectively. The cumulative percentages are 99.0 (CDA1) and 100.0 (CDA2). In case of Fig. 1B, the first function accounts for 87.9%, the second accounts for 11.0%, the third accounts for 0.7%, while the fourth function accounts for 0.4% of the discriminating ability of the discriminating variables. The canonical correlation values are 0.999, 0.994, 0.916 and 0.851. The cumulative percentages are 87.9% (CDA1), 98.9% (CDA2), 99.6% (CDA3), and 100% (CDA4).

Differences were found in the concentration of elements considering the year of collection, which is indicated in Fig. 1C, D through the example of three macro- (K, Mg, and Ca) and three micro- (Sr, Ba, and Cr) elements. The K and Cr content of the samples showed a strong negative correlation with the age, *p* = 0.00485 for K and *p* = 0.00034 for Cr. At the same time, the Ca content has shown a slightly increasing tendency over the years (*p* = 0.037448). Potassium is considered one of the main cations present in wine samples. Its level is dominantly affected by the botanical origin of the berries, soil, climatic conditions and less markedly wine-making procedures. In our previous study, considering the elemental profiling of historical honey samples, the K level remained relatively unchanged considering the years of collection^33,34^.

Since it is one of the most important plant macronutrients, its level is managed by agricultural practices such as fertilization regimes and soil management. These processes can ultimately impact its concentration in grapes and subsequently in wines^45^. While the issues regarding the sustainability of nitrogen and phosphorus are more discussed, potassium is also an element in danger. Higher level is usually extracted globally by harvesting compared to the amount added by fertilizing. However, higher than required level of potassium also has an adverse effect on wine^46^. Grape berries act as a sink for K, and its elevated concentration can lead to the loss of tartaric acid (precipitates as potassium bitartrate). Due to this phenomenon, a more difficult pH adjustment is required in the wine-making procedure, which significantly increases the production costs^47,48^. Since product quality is the bottleneck of market competition, balancing the K requirements for optimal growth and product parameters are essential. Still, there are areas where the mechanism of K accumulation in grape berries lacks information^47^. The analysis of old wine samples gives the possibility of long-term monitoring for K content. Together with other soil-related agricultural data and production information, it can support the further understanding and optimization of the desired K levels as well as the establishment of the related fertilizing program.

In contrast to K, the accumulation of Ca takes place in the early stage of growth. Since it is a phloem-immobile nutrient, only a limited amount has access directly to the berry^49^. Calcium is also an important plant nutrient and strong antagonism regarding the Ca-K levels are described in the literature: higher K concentration in grape berries usually results in a lower amount of Ca and the other way around. Garcia et al. studied the effect Ca-K ratios hydroponically on cation levels and the growth performance of grapes. It was found that the Ca supplementation resulted in 30% decreased K concentration in leaf blades and petioles. Liming thus affects K uptake and elevates acidity^50^. Our results reflect this phenomenon in a long-term scale: K showed a decreasing pattern while Ca a slightly increasing one over the years of production.

The level of Cr negatively correlated with the year of origin, suggesting a change in production processes. This element may originate partly from the vineyard soil, it is also widely utilized in stainless steel production, serving as an electroplated coating to combat oxidative deterioration. Thus, its presence in wine can be related to the manufacturing procedures, and also the chromium oxides can exit wine from glass pigments. Cabrera-Vique et al. (1997) illustrated that chromium content elevated with bottle aging across different vintages of red wine crafted through identical methods, sourced from the same vineyard and winery^51,52^. The extraction of chromium from foodstuffs is pH dependent—it increases under acidic conditions. Its concentration is thus suggested to be used as a proxy for manufacturing authentication^41^.

### Influence of rainfall on elemental composition

**Fig. 2:**
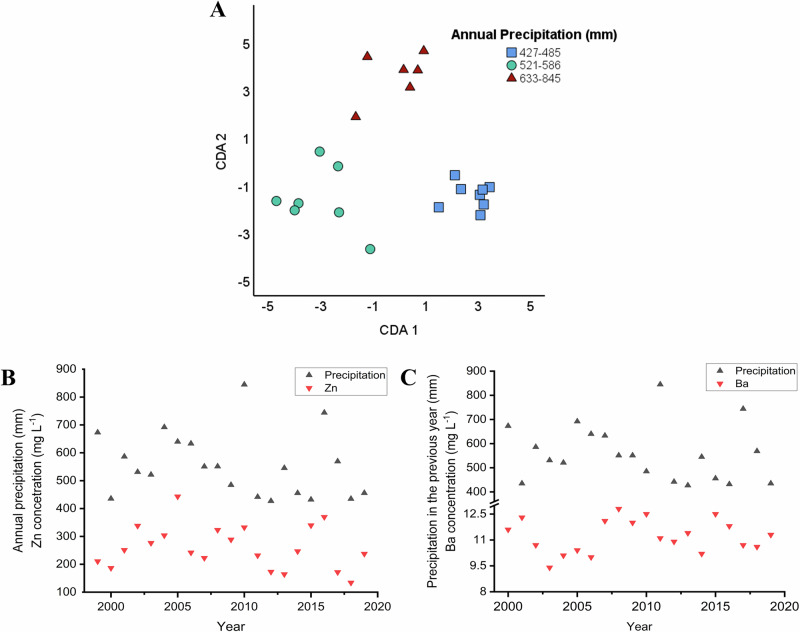
CDA biplot showing group separation of wine samples by annual precipitation. **A** CDA biplot graph of Tokaji aszú type wines differentiated based on the amount of precipitation recorded in their year of origin. (Samples are grouped based on the amount of precipitation as follows: 427–456 mm, 485–551 mm, 568–640 mm, 673–845 mm). Axes represent canonical functions accounting for variance in elemental composition. **B** Interaction between the yearly precipitation and the Zn content of aszú samples. **C** Correlation between the previous year’s precipitation and the Ba content of aszú samples. Precipitation values are expressed in mm, while elemental concentrations are given in mg L^−1^. Meteorological data received from www.met.hu^72^.

The composition of wines is significantly influenced by various environmental factors, including changing climatic trends and weather conditions^53–56^. To investigate the impact of annual rainfall—a key environmental factor—on the elemental composition of wine, a multivariate statistical analysis was carried out. For this purpose, wine samples were grouped based on the amount of precipitation recorded in their year of origin: 427–456 mm, 485–551 mm, 568–640 mm, 673–845 mm (Fig. 2A).

The CDA biplot in Fig. 2A indicates a prominent separation between the wines produced in rainier years compared to those made in droughtier ones due to their different elemental content. The first function accounts for 79.0%, the second accounts for 14.0%, while the third accounts for 6.1% of the discriminating ability of the discriminating variables. The canonical correlation values are 0.988, 0.940, and 0.869. The cumulative percentages are 79.0% (CDA1), 93.9% (CDA2), and 100.0% (CDA3). The separation of the groups is supported by the positive correlation of the samples’ Zn content with the total annual precipitation (*p* = 0.03592), as shown in Fig. 2B. Additionally, the statistical analysis indicates that the Ba content of the samples is negatively correlated with the previous year’s precipitation (*p* = 0.00881) indicated in Fig. 2C. Furthermore, the Ca content is negatively correlated with the rainfall received two years earlier (*p* = 0.04164). The observed tendencies can be explained by the fact that soil cultivation practices and rainfall characteristics, such as its intensity and quantity, influence the leaching of certain elements, including zinc, from vineyard soils^39,57^. This phenomena directly affects the elemental content of grapes and wine and is strongly influenced by the soil structure and clay type prevalent in the Tokaj region^58^.

### Influence of sunshine hours and temperature on elemental composition

**Fig. 3:**
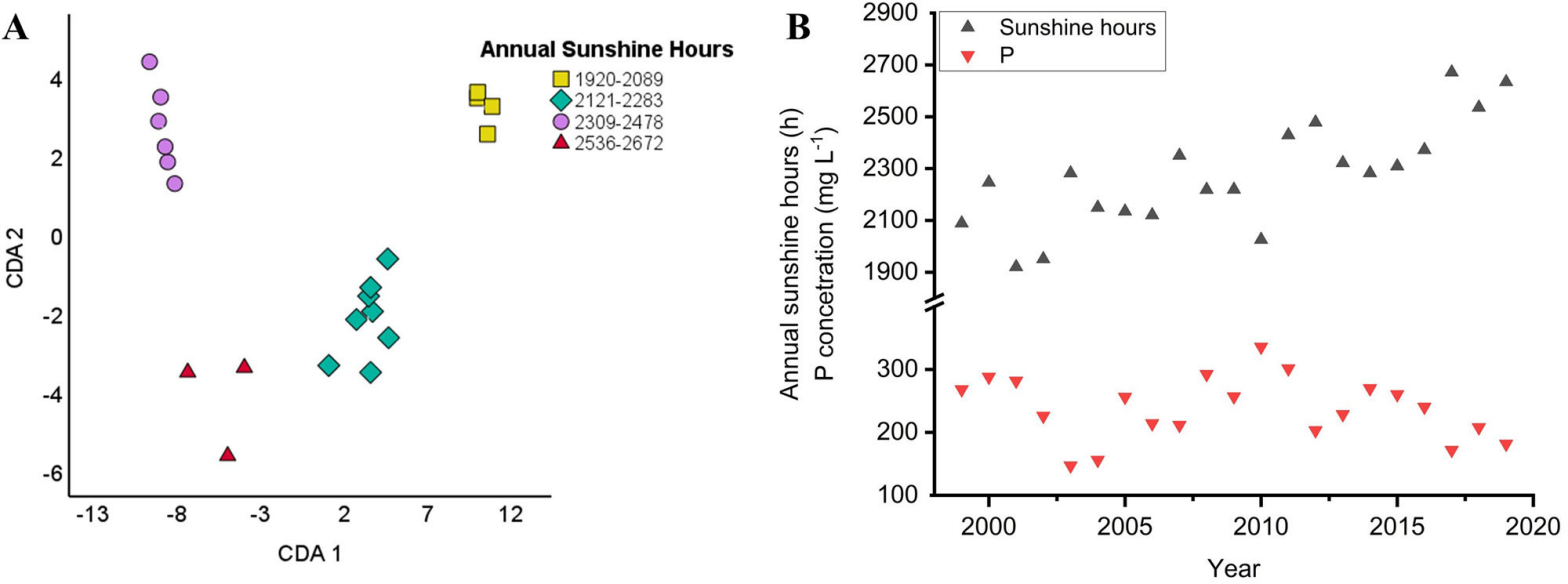
CDA biplot showing group separation of wine samples by annual sunshine hours. **A** CDA biplot graph of Tokaji aszú type wines differentiated based on the number of sunshine hours recorded in their year of origin. (Samples are grouped based on the number of sunshine hours as follows: 1920–2089, 2121–2283, 2309–2478, 2536–2672). Axes represent canonical functions accounting for variance in elemental composition. **B** Correlation between the annual sunshine hours and the P content of aszú samples. Meteorological data received from www.met.hu^72^.

In order to investigate whether the annual number of sunshine hours also has a significant effect on the elemental composition of the analysed samples, additional statistical tests were conducted. The wines were grouped for the multivariate statistical analysis based on the number of sunshine hours recorded in their year of origin: 1920–2089, 2121–2283, 2309–2478, 2536–2672 (Fig. 3A). As indicated in the Fig. 3A CDA biplot, a separation was found considering the elemental data of wines produced in years with a higher number of sunshine hours compared to those with fewer. The first function accounts for 83.3%, the second accounts for 13.3%, while the third accounts for 3.4% of the discriminating ability of the discriminating variables. The canonical correlation values are 0.992, 0.954, and 0.851. The cumulative percentages are 83.3% (CDA1), 96.6% (CDA2), and 100.0% (CDA3).

The separation of the groups is supported by the fact that the concentration of several elements tends to vary with the number of sunny hours. Statistics show a significant negative correlation between the total annual sunshine hours and the P (Fig. 3B), Cr, Sr, and Zn content of the samples, for which the *p* values are *p* = 0.04786, *p* = 0.00144, *p* = 0.02139, *p* = 0.02624. The explanation for the observed phenomenon is that when plants grow more intensely and have an enhanced photosynthetic activity in response to higher amounts of sunlight, they tend to reallocate their available nutrients to areas where they are most needed to support other physiological processes^59,60^.

**Fig. 4:**
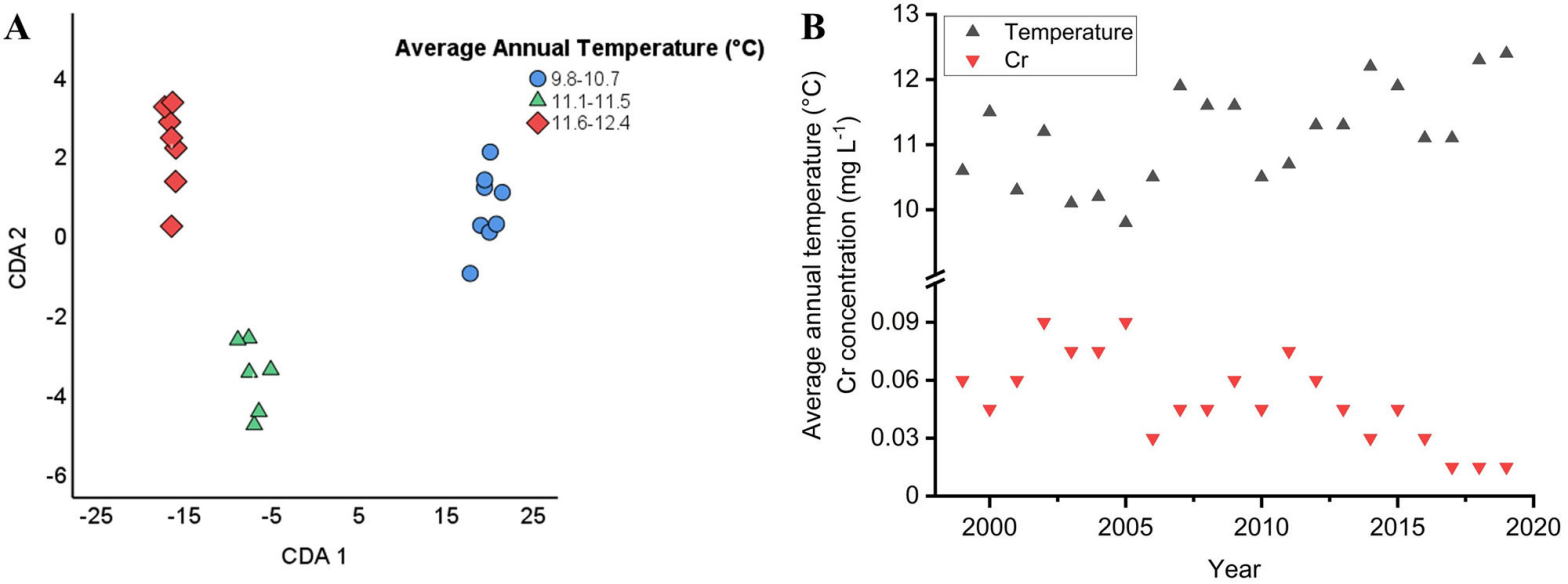
CDA biplot showing group separation of wine samples by average annual temperature. **A** CDA biplot graph of Tokaji aszú type wines differentiated based on the average temperature in their year of origin. (Samples are grouped based on the average annual temperature as follows: 9.8–10.7 °C, 11.1–11.5 °C, 11.6–12.4 °C). Axes represent canonical functions accounting for variance in elemental composition. **B** Correlation between the average annual temperature and the Cr content of aszú samples. Temperature values are expressed in °C, while elemental concentrations are given in mg L^−1^. Meteorological data received from www.met.hu^72^.

Multivariate statistical analysis was carried out on the elemental compositions of the wines also by making groups based on the average annual temperature in their year of origin: 9.8–10.7, 11.1–11.5, 11.6–12.4 (Fig. 4A). The CDA biplot of Fig. 4A illustrates the separation considering the elemental data of wines produced in years with higher average annual temperature compared to those with lower. The first function accounts for 97.9%, the second accounts for 2.1% of the discriminating ability of the discriminating variables. The canonical correlation values are 0.998 and 0.930. The cumulative percentages are 97.9% (CDA1) and 100.0% (CDA2). The separations are supported by the finding that the average annual temperature correlates negatively with the Cr content of samples (*p* = 0.00233), as shown in Fig. 4B.

Blotevogel et al. (2019) quantitatively investigated those elements that are the most applied tracers in geographical origin—namely Ba, Ca, Mg, Mn, and Sr—in West European wines. In their statistical analysis, weather conditions explained 23.4% of the variance for the studied elements. Higher concentrations of Ba and Mn were associated with increased summer rainfall, while elevated Sr levels correlated with higher temperatures^61^. Greenough et al. (2005) previously linked high Sr contents in wines to arid conditions, highlighting also the effect of the presence and absence of precipitation^62^. Blotevogel et al. (2019) draw similar conclusions to the present study, explaining the phenomena by drought stress that, in their case, decreased Ba and Mn concentrations and increased Sr concentrations. The phenomenon is likely due to evaporation processes and the accumulation of carbonate salts in typical dry-climate soils. They also found a correlation between Mn and Ba concentrations and higher precipitation, particularly during the summer. The authors explained it by the greater mobility of these elements in wet soils with low redox potential or by the increased acidification of soils with higher rainfall^61^.

### Results of the FTIR analysis

**Fig. 5:**
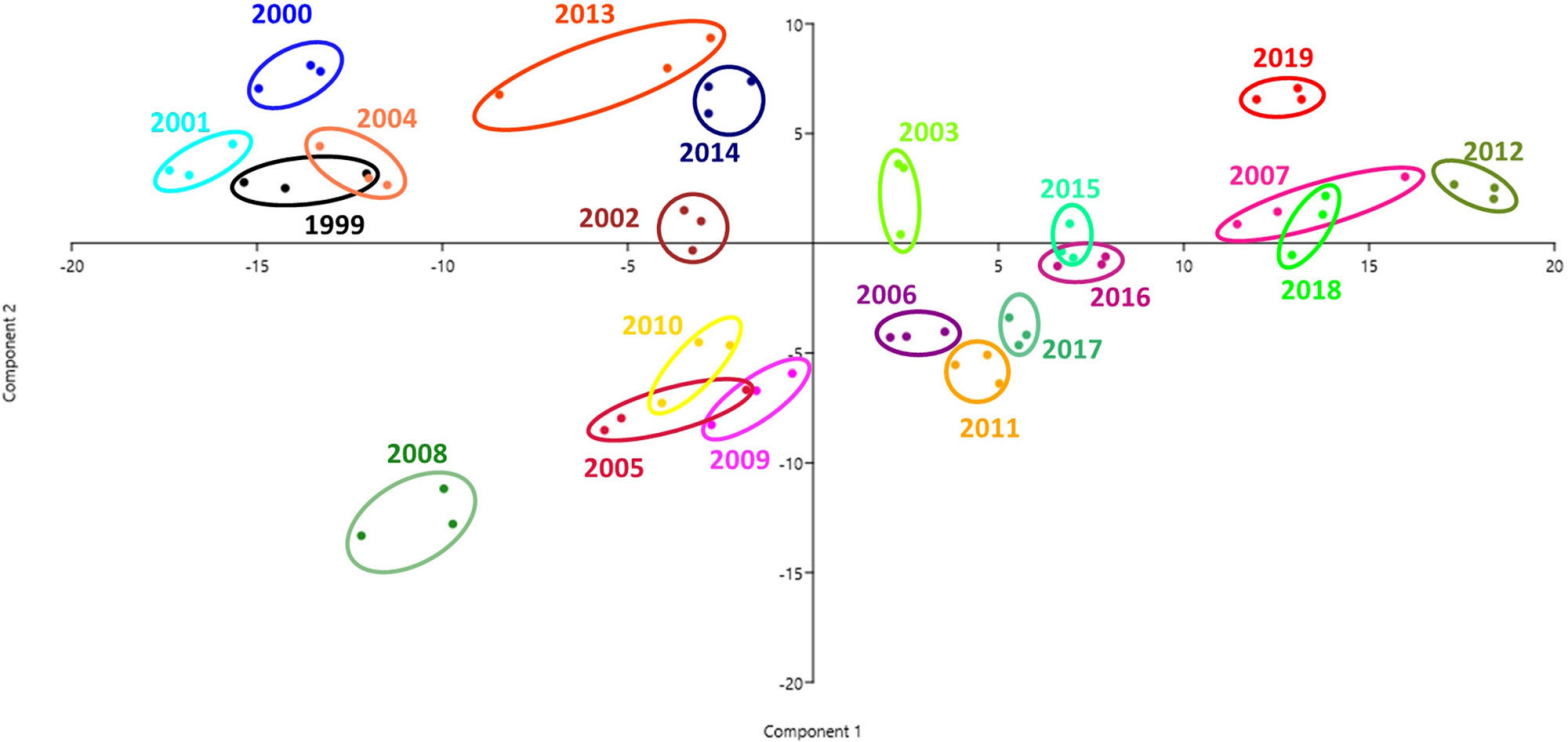
The PCA graph based on the intensity – wavenumber data pairs obtained by the FTIR analysis of the wine samples. Replicates from the identical samples have been marked in the same color for ease of visibility, and the vintage has been indicated alongside the groups.

The IR spectra of the 21 wine samples was obtained by FTIR conducting at least three repetition out of each. Although the instrument performed measurements in a greater range of spectra (4000–650 cm^−1^), we focused our analysis on the fingerprint region (600–1800 cm^−^¹), indicated in Supplementary Fig. 1, as this spectral range contains a complex array of absorption bands arising from various molecular vibrations, including those of carbohydrates, organic acids, phenolic compounds, and amino acids—key constituents of wine. These bands are often highly specific and sensitive to subtle compositional changes, making the fingerprint region particularly suitable for differentiating samples based on vintage or production-related variations. Furthermore, this region has been widely reported in the literature as the most informative part of the mid-infrared spectrum for wine profiling^63–65^. Principal component analysis was used to visualize differences between samples. Given that only one wine sample per year was available for analyses, the spectral replicates do not provide independent observations. Thus, PCA results are considered exploratory, and no validation set was used to confirm classification. The PCA graph of Fig. 5. indicates that certain years produced wines with highly similar chemical composition, e.g., 2015 and 2016, although differences are also noticeable. The visual clustering may be explained by the environmental factors of the particular years. For instance, the vintages 2007, 2012, 2018, and 2019 being arranged around one another can be attributed to the fact that they had summers with above-average temperatures, resulting in a higher sugar content in berries. This finding is also underlined by the greater intensities recorded in the range 1000–1150 cm^−1^ where the peaks belonging to the carbohydrates (glucose, fructose, and oligosaccharides) can be found.

FTIR spectroscopy already has a widespread application in the wine industry, utilizing the easy-to-use possibility offered by the portable instruments. The fast and relatively cost-effective technique can be used in product monitoring and process control, while together with multivariate statistical it plays an important part in authenticity and traceability efforts^66–68^. Although minor clustering tendencies were observed between older and newer vintages in the PCA space, these are not statistically validated and may reflect minor compositional trends. However, expanding the sample number may lead to a clearer separation of old wines from different vintages based on their composition^69^. This study is among the first to explore the application of this method to old *aszú* wines, indicating its potential to understand the influence of environmental factors on wine quality. However, future studies incorporating multiple bottles per vintage and external validation sets are required for more robust modeling.

## Discussion

This study highlights the significant role that elemental profiling can play in understanding the complex interplay between environmental factors, agricultural practices, and the chemical composition of Tokaji aszú wines. By analyzing wine samples spanning two decades, we have demonstrated that these wines serve as valuable archives of historical environmental data, reflecting long-term trends in climate, soil management, and viticulture practices, providing valuable insights into the various vintage-specific influences. Tracking the dynamics of elements is crucial, particularly given their significance as vital plant nutrients or considering them as potential toxic agents. Understanding the composition of old wines allows us to predict future agricultural outcomes and gain a deeper understanding of external factors impacting wine production. Our findings reveal distinct correlations between elemental content and key environmental variables such as precipitation, temperature, and sunshine hours, underscoring the influence of these factors on the quality and characteristics of wine. Similar approaches have been applied in wine studies aiming to link terroir or vintage-specific effects to wine chemistry, and our results support the notion that such climatic classifications may contribute valuable context when interpreting analytical data. Furthermore, the application of advanced analytical techniques like ICP-OES and FTIR spectroscopy has proven effective in distinguishing between vintages and identifying subtle shifts in wine composition. These techniques not only enhance our understanding of historical wine production but also provide a robust framework for assessing the authenticity and origin of wines. The implications of this research extend beyond the preservation of traditional wine-making practices. By providing a clearer understanding of how environmental factors influence wine composition, this study contributes to the development of more informed and sustainable viticultural strategies. For viticulturists and winemakers, such data-driven insights can support evidence-based decision-making regarding varietal selection, harvest timing, and soil or nutrient management, all of which are increasingly important under changing climatic conditions. The ability to trace vintage-specific influences over decades can also aid in anticipating how future environmental scenarios may affect grape quality and wine composition. From a sustainability perspective, these findings may inform adaptive agricultural practices that aim to maintain wine quality while minimizing environmental impact. Additionally, integrating elemental profiling into vineyard monitoring programs could serve as an early-warning system for shifts in environmental conditions or soil degradation, contributing to more resilient and climate-smart viticultural systems. As the wine industry faces the challenges of climate change, such insights are essential for maintaining the quality and heritage of renowned wine regions like Tokaj. In conclusion, the elemental profiling of Tokaji aszú wines offers a powerful tool for both historical analysis and future planning. This comprehensive analysis underscores the importance of monitoring and analyzing both the chemical and elemental profiles of wines to ensure quality, authenticity, and sustainability in the wine industry.

## Methods

### Wine samples

**Fig. 6:**
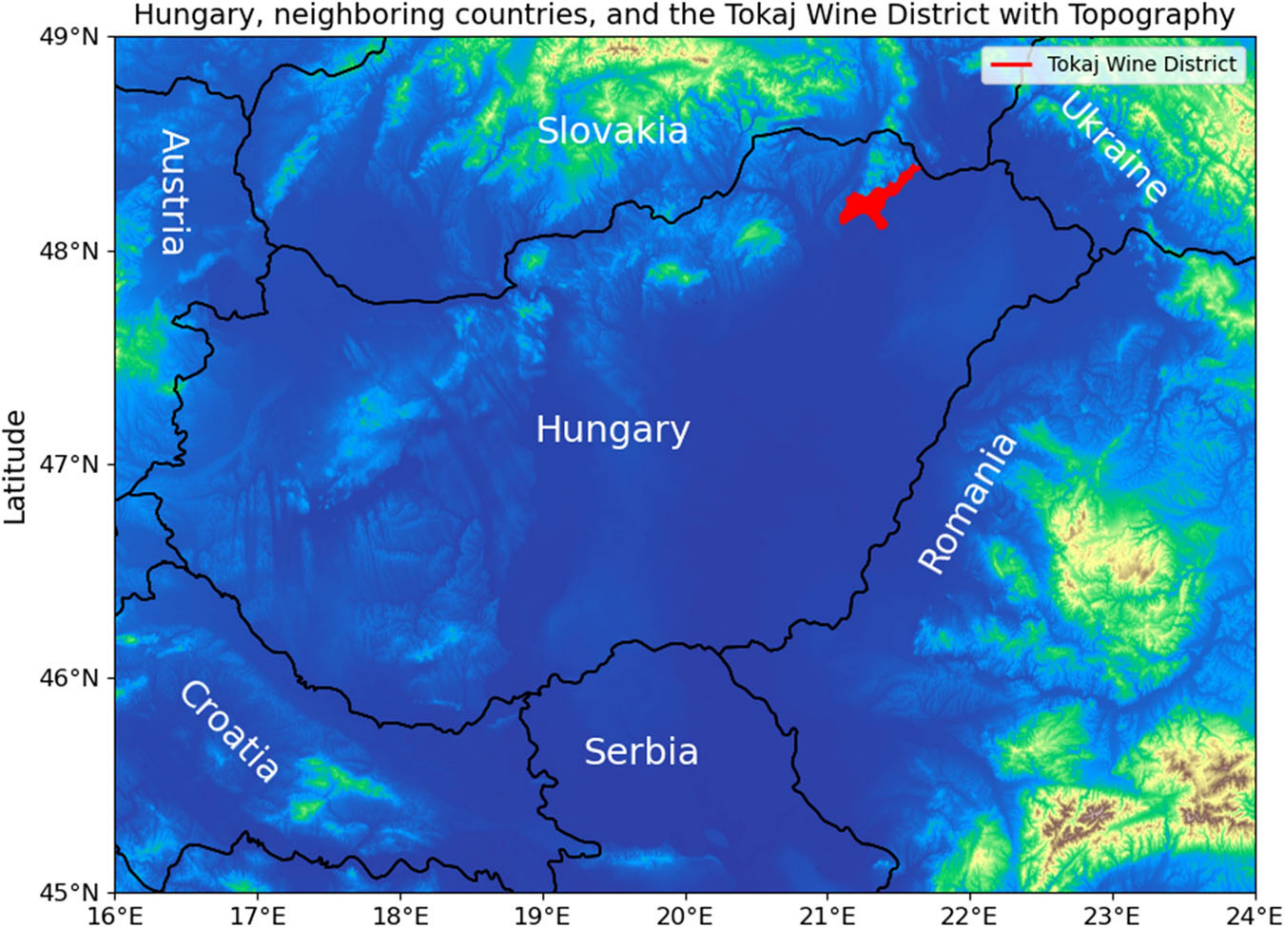
The location of tokaj wine district. Hungary, it’s neighboring countries, and the Tokaj wine region.

Twenty-one Tokaji aszú samples were obtained from the Tokaj-Hétszőlő winery, located at the foothills of the Zemplén Mountains in the Tokaj Wine Region (Fig. 6). This region, renowned for its rich viticultural history, is the second oldest legally protected vineyard zone in the world and was designated as a World Heritage Site by UNESCO in 2002^70^. The analysed wine samples were produced between 1999 and 2019 annually, being the longest time interval in which the elemental composition of aszú type wines were ever investigated based on literature data. One sample per year was analyzed from 1999 to 2019, totaling 21 5 puttonyos Tokaji aszú samples of different vintages. In previous research, Varga et al. confirmed the age of these wine samples using accelerator mass spectrometry (AMS) based radiocarbon dating^71^.

### Sample preparation

Each wine sample (1.00 mL) was measured into 50.0 ml volume glass beakers and placed on an electric hot plate with a temperature of 100 °C to receive dry residue. Dried samples were digested by atmospheric wet digestion with the mixture of 7.00 mL 65% (m/m) HNO_3_ (reagent grade, Scharlau) and 1.00 mL 30% (m/m) H_2_O_2_ (reagent grade, Merck) at 100–120 °C for 4 h. Digested samples were transferred without loss into volume-calibrated plastic centrifuge tubes and diluted up to a volume of 15.00 mL with 0,1 M HNO_3_ prepared in ultrapure water (Synergy UV Millipore). Solutions were kept at room temperature prior to further elemental analysis.

### ICP-OES analysis

The elemental concentration of the samples was determined by inductively coupled plasma optical emission spectrometry (ICP-OES 5110 Vertical Dual View, Agilent Technologies). Measurements were performed using autosampler (Agilent SPS4), Meinhard^®^ type nebulizer and double pass spray chamber as well as a five-point calibration procedure was applied (ICP VI, Merck). The ICP-OES operating conditions and measurement parameters are indicated in Tables 1 and 2, respectively.

**Table 1:** ICP-OES conditions I

Common conditions	
Replicates	3
Pump speed	12 rpm
Uptake time	15 s
Rinse time	30 s
Read time	10 s
RF power	1.20 kW
Stabilization time	10 s
Nebulizer flow	0.70 L min^−1^
Plasma flow	12.0 L min^−1^
Aux flow	1.0 L min^−1^
Viewing height	8 mm

**Table 2:** ICP-OES conditions II

Element	Wavelength (nm)	Viewing mode	Stabilization time (s)
Al	396.152	axial	10
B	249.772	axial	10
Ba	455.403	axial	10
Ca	317.933	axial	10
Cr	267.716	axial	10
Cu	324.754	axial	10
Fe	238.204	axial	10
K	766.491	axial	10
Mg	280.270	axial	10
Mn	257.610	axial	10
Na	589.592	axial	10
Ni	231.604	axial	10
P	213.618	axial	10
Pb	220.353	axial	10
S	180.669	axial	10
Sr	407.771	axial	10
Zn	213.857	axial	10

Standard solutions of the macro elements (Ca, K, Mg, Na) were prepared from mono element spectroscopic standards with a concentration of 1000 mg L^−1^ (Scharlau), while of the micro elements (Al, B, Ba, Cr, Cu, Fe, Mn, Ni, P, Pb, S, Sr, Zn) from the multi element spectroscopic standard solution of 1000 mg L^−1^ (ICP IV, Merck). In each case, a five-point calibration process was used, for which standard solutions were diluted with 0.1 M HNO_3_ prepared in ultrapure water. Since there is no available Certified Reference Material (CRM) for aszú wine, the spike technique was used to validate the ICP-OES method. Commercially available aszú wines were purchased and digested in the same way as described for the collected sample series. Yttrium (1000 mg L^−1^, Merck) and lithium (1000 mg L^−1^, Merck) standard solutions were applied as internal standards in the final concentrations of 1 and 5 mg L^−1^ to verify the sample pre-treatment and the measurement processes (Y was added to the samples prior to the digestion step while Li was added prior to the elemental analysis). Solutions were measured by ICP-OES methods, and recoveries were evaluated. The received recoveries were within 5% for all samples. The relative standard deviation (RSD) of triplicate ICP-OES measurements was also below 5% for all elements.

### FTIR analysis

Fourier transform infrared spectroscopy (FTIR) was used to determine the infrared (IR) spectra of the wine samples. The spectrometer (Cary 660, Agilent Technologies) being applied is operated by the software MicroLab PC. Measurements were carried out using the attenuated total reflection (ATR) mode. Samples were analysed straight after homogenization, without the addition of any chemical reagents. The crystal of the instrument was accurately cleaned with ultrapure water and ethanol (reagent grade, Merck) before performing the analysis and between samples. The spectra were measured in a mid-infrared region (4000–650 cm^−1^) using a nominal recording resolution of 4 cm^−1^ in at least three individual replicates. The software made 16 background and 128 sample scans in case of each replicates. Happ-Genzel apodization was used to obtain the appropriate balance between ripple size and resolution, coupled with Mertz phase correction. Spectral replicates for FTIR showed minimal variation, with mean intra-sample spectral deviation <1% across the fingerprint region. Post-analysis, the wavelength and intensity data pairs were exported for statistical evaluation. The spectral analysis was carried out similarly to our previous study^33^ using the Unscrambler Xv10.3 software package. For smoothing the FTIR spectra and for derivatization, the Savitzky-Golay method was applied. The Multiplicative Scatter Correction (MSC) was used to compensate for additive and/or multiplicative effects in the spectral data.

### Data evaluation

Elemental concentration data were compared using ANOVA (Analysis of Variances), where significant differences were estimated by Tukey’s multiple response test, and the homogeneity of variances was confirmed by a Levene test. Differences were considered to be significant if the *p* value was <0.05. Canonical discriminant analysis (CDA) was applied to separate the concentration data according to the year of sample origin, the amount of yearly rainfall, the annual sunshine hours and the average annual temperature. The SPSS software package was used (SPSS Statistics IBM 28.0.0.0) to perform ANOVA and CDA tests on elemental analytical results. The FTIR results were subjected to principal component analysis (PCA) using the software Past 4.03. For CDA of the elemental analysis results data matrix comprised 21 samples × 16 elements. For PCA of the FTIR spectra, the matrix consisted of 105 spectra (5 replicates × 21 samples) × ~300 variables corresponding to the 600–1800 cm^−^¹ fingerprint region. Spectral data were baseline corrected and normalized prior to PCA using standard normal variate (SNV) transformation.

In order to assess the potential impact of vintage-related environmental factors on wine composition, the samples were grouped according to harvest year and selected climatological parameters, namely precipitation, sunshine duration, and mean growing season temperature. This approach aimed to explore whether macroclimatic conditions in the vineyard during the grape ripening period could be reflected in either the elemental content or the FTIR spectral profiles of the resulting wines. Yearly meteorological data for the Tokaj wine region were obtained from regional databases and classified into categories based on significant deviations from long-term averages. Groupings were determined based on natural breaks in the distribution of recorded values as well as agronomic relevance (e.g., thresholds commonly used in viticultural meteorology to distinguish between dry, moderate, and wet years). This grouping strategy, although limited by the small number of samples per category, provided a preliminary framework for investigating whether climatic variability could be linked to detectable analytical differences.

## Supplementary information

Supplementary Infomation

## Data Availability

The datasets used and/or analysed during the current study are available from the corresponding author on reasonable request.
