# Supplementary material for: Long term compositional profiling of historical tokaji aszú wines
Source: NPJ Sci Food. 2025 Jun 14;9:101. doi: 10.1038/s41538-025-00468-x (PMC12166034; doi:10.1038/s41538-025-00468-x)
Supplement: Supplementary file 1 — Supplementary Infomation [file 41538_2025_468_MOESM1_ESM.docx]

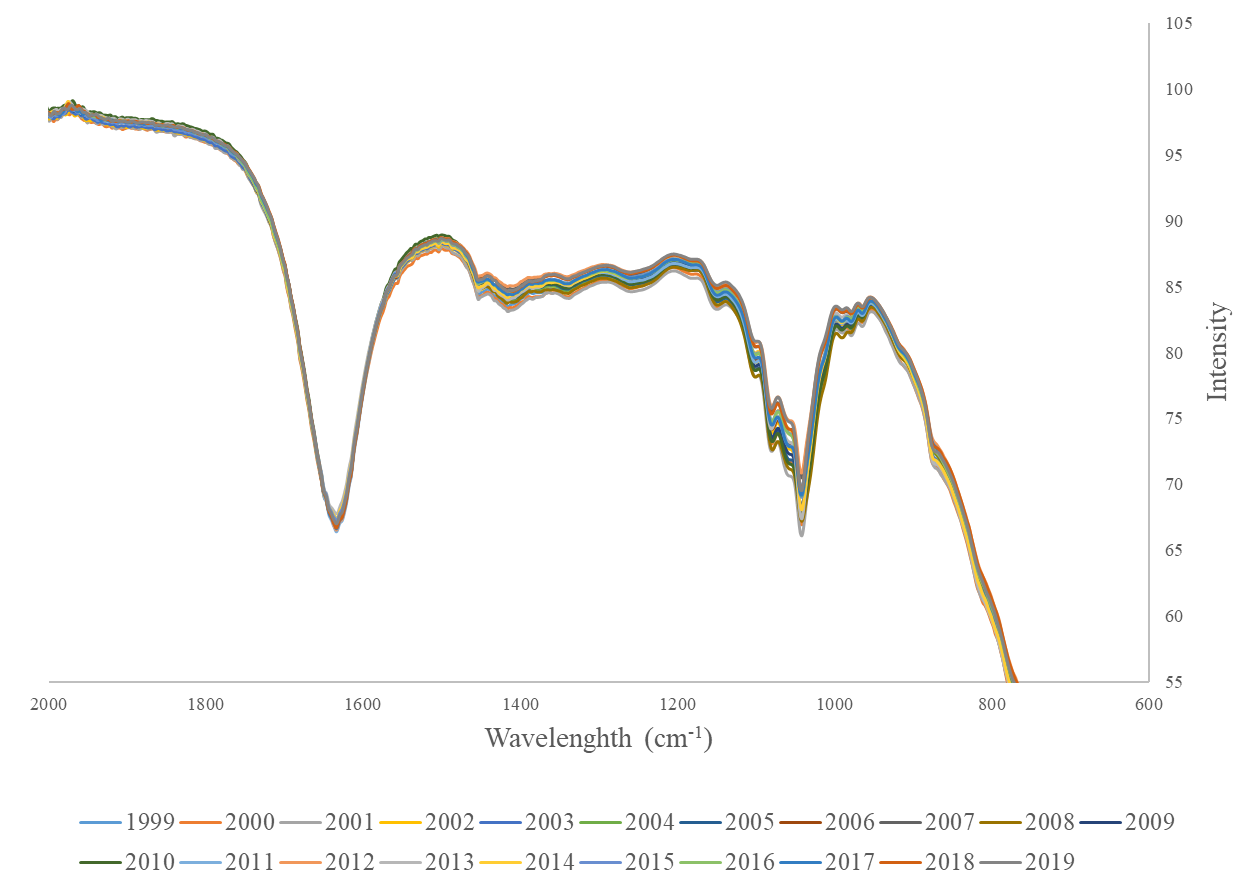


**Suppl. Fig. 1.** FTIR spectra of the aszú wines focusing on the fingerprint region (600 – 1800 cm^-1^) (Intensities are plotted as the average of the parallel measurements per vintage (n=3). Vintages are indicated with different colours.)
